# Glioma Grading by Integrating Radiomic Features from Peritumoral Edema in Fused MRI Images and Automated Machine Learning

**DOI:** 10.3390/jimaging11100336

**Published:** 2025-09-27

**Authors:** Amir Khorasani

**Affiliations:** 1 Medical Image and Signal Processing Research Center, School of Advanced Technologies in Medicine, Isfahan University of Medical Sciences, Isfahan 81746-73461, Iran; a.khorasani@resident.mui.ac.ir or amir69k@yahoo.com; Tel.: +98-31-37923865; 2 Department of Bioimaging, School of Advanced Technologies in Medicine, Isfahan University of Medical Sciences, Isfahan 81746-73461, Iran

**Keywords:** radiomic, machine learning, magnetic resonance imaging, image fusion, glioma

## Abstract

We aimed to investigate the utility of peritumoral edema-derived radiomic features from magnetic resonance imaging (MRI) image weights and fused MRI sequences for enhancing the performance of machine learning-based glioma grading. The present study utilized the Multimodal Brain Tumor Segmentation Challenge 2023 (BraTS 2023) dataset. Laplacian Re-decomposition (LRD) was employed to fuse multimodal MRI sequences. The fused image quality was evaluated using the Entropy, standard deviation (STD), peak signal-to-noise ratio (PSNR), and structural similarity index measure (SSIM) metrics. A comprehensive set of radiomic features was subsequently extracted from peritumoral edema regions using PyRadiomics. The Boruta algorithm was applied for feature selection, and an optimized classification pipeline was developed using the Tree-based Pipeline Optimization Tool (TPOT). Model performance for glioma grade classification was evaluated based on accuracy, precision, recall, F1-score, and area under the curve (AUC) parameters. Analysis of fused image quality metrics confirmed that the LRD method produces high-quality fused images. From 851 radiomic features extracted from peritumoral edema regions, the Boruta algorithm selected different sets of informative features in both standard MRI and fused images. Subsequent TPOT automated machine learning optimization analysis identified a fine-tuned Stochastic Gradient Descent (SGD) classifier, trained on features from T_1_Gd+FLAIR fused images, as the top-performing model. This model achieved superior performance in glioma grade classification (Accuracy = 0.96, Precision = 1.0, Recall = 0.94, F1-Score = 0.96, AUC = 1.0). Radiomic features derived from peritumoral edema in fused MRI images using the LRD method demonstrated distinct, grade-specific patterns and can be utilized as a non-invasive, accurate, and rapid glioma grade classification method.

## 1. Introduction

Gliomas are the most common primary brain tumors and are classified into four grades by the World Health Organization (WHO), based on their malignancy and biological behavior, which reflects the biological aggressiveness of glioma tumors [1,2]. Low-grade gliomas (LGGs, WHO Grades I and II) are typically characterized by slower growth and diffuse infiltration into surrounding brain parenchyma [3]. On the other hand, high-grade gliomas (HGGs, WHO Grades III and IV), most notably the devastating glioblastoma (GBM), exhibit rapid, invasive growth, pronounced angiogenesis, and central necrosis [4]. This aggressive phenotype is not merely a histological distinction but is manifested in their interaction with the microenvironment, particularly through the formation of extensive vasogenic edema and profound blood–brain barrier disruption [5,6,7]. The grading system is crucial for determining prognosis and guiding treatment strategies. Gliomas, particularly GBMs, exhibit distinct regions, including edema, necrosis, and active tumor areas, each with unique characteristics and implications for diagnosis and treatment [8].

The gold standard for glioma grading is histopathological evaluation of biopsy specimens based on cellular characteristics under a microscope [9]. The procedure is invasive, with a risk of sampling error, and is a time-consuming process. Histopathological evaluation can be subjective, leading to variability in diagnosis among different pathologists [10]. These limitations underscore the critical need for reliable, non-invasive, fast, and accurate prognostic tools. In this context, quantitative radiomic analysis extracted from magnetic resonance imaging (MRI) sequences has been used in previous studies [11,12,13]. Radiomics is a computational methodology that extracts a vast number of quantitative, sub-visual features from standard medical images to characterize tissue and predict clinical outcomes [14]. Previous radiomics studies for glioma grading have focused on the extraction of features from the whole tumor or active core regions. Recent advances now leverage the peritumoral edema radiomic feature [15], a region present in both LGGs and HGGs [16,17], unlike the enhancing core, which is primarily seen in HGGs [18,19]. Therefore, this study utilized radiomic features extracted from the peritumoral edema region for the classification of glioma grades.

Most studies in the field of glioma grade classification using radiomic features have focused solely on the use of single MRI image features or multiparametric MRI features [20,21,22,23]. Recently, researchers have shown an increased interest in using image fusion methods for fusing MRI image weights for glioma grade classification [24,25]. 

Image fusion integrates complementary information from different imaging modalities into a single image. This combined image provides a more comprehensive view, highlighting tumor and edematous features more effectively than individual images. Image fusion techniques, such as the Laplacian Re-decomposition (LRD), have demonstrated high performance in producing high-quality fused images compared to other fusion methods [26,27]. The aim of this study has therefore been to assess the use of radiomic features in peritumoral edema regions of fused MRI images for improving glioma grade classification performance.

## 2. Materials and Methods

**Figure 1:**
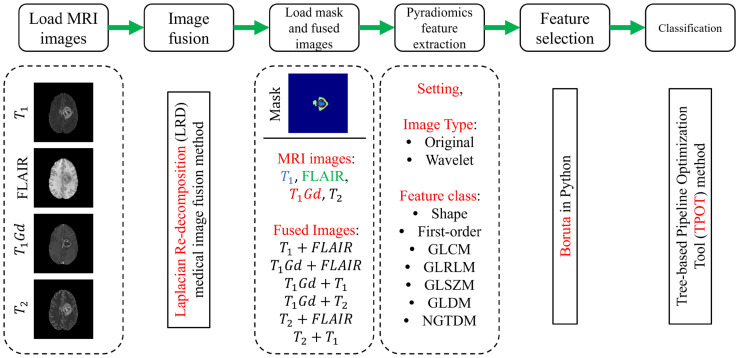
Study workflow for radiomic feature extraction and glioma grade classification from MRI scans and fused MRI images. The process utilized MRI scans, fused MRI images, and corresponding edema region masks from the BraTS training dataset. Radiomic features were extracted from both original and fused images using the PyRadiomics library, followed by feature selection and classification with Boruta and Tree-based Pipeline Optimization Tool (TPOT) methods, respectively. Abbreviations: GLCM: Gray-Level Co-occurrence Matrix, GLRLM: Gray-Level Run Length Matrix, GLSZM: Gray-Level Size Zone Matrix, GLDM: Gray-Level Dependence Matrix, NGTDM: Neighborhood Gray-Tone Difference Matrix.

The study workflow, depicted in Figure 1, summarizes the methodological steps from MRI image loading to glioma grade classification. The study was conducted in several key stages: loading the MRI images, image fusion, creation of the edema region mask, radiomics feature extraction and PyRadiomics setting, feature selection, and finally, glioma grade classification.

Ethical approval for this study was granted by the research ethics committee of Isfahan University of Medical Sciences, Isfahan, Iran, with the ID IR.MUI.DHMT.REC.1403.213.

### 2.1. MRI Images

This study utilized the Multimodal Brain Tumor Segmentation Challenge 2023 (BraTS 2023) dataset (https://www.synapse.org/Synapse:syn64952532, Accessed on 10 January 2024), a widely recognized benchmark for brain tumor analysis. The dataset comprises multi-institutional preoperative multimodal MRI scans (T_1_, T_1_Gd, T_2_, and FLAIR sequences) with corresponding manual segmentations of glioma tumors. This dataset consists of LGGs and HGGs. These segmentations, annotated by expert neuroradiologists, delineate tumor subregions, including the enhancing tumor, peritumoral edema, and necrotic/core regions. The BraTS 2023 training set includes preprocessed, skull-stripped, and co-registered volumes, ensuring standardized spatial resolution (1 mm^3^ isotropic) and alignment across modalities. Ground truth masks enable robust radiomic feature extraction and model training, while the dataset’s diversity in tumor grade and size supports generalizability.

### 2.2. Image Fusion

For this study, the LRD medical image fusion was used to fuse the MRI image weights. The LRD medical image fusion approach was selected based on prior studies demonstrating its ability to generate high-quality fused images and its effectiveness in glioma grade classification [24,26]. The MRI image fusion process, implemented in MATLAB 2019a, followed the LRD algorithm proposed by Li et al. [27]. Briefly, the key steps included the following: (1) Gradient Domain Image Enhancement (GDIE) to generate enhanced images (H_A_/H_B_), (2) Laplacian Pyramid Transform (LP) to decompose images into low- (G_A_/G_B_) and high-frequency (L_A_/L_B_) sub-band images, (3) Decision Graph Re-decomposition (DGR) to create overlapping (O_A_/O_B_) and non-overlapping (N_A_/N_B_) domains, and (4) fusion rules (local energy maximum (LEM), overlapping domain (OD), non-overlapping domain (NOD), and inverse re-decomposition scheme (IRS)) to reconstruct the final image.

When employing the LRD framework for image fusion, several key settings and rules are crucial for achieving optimal results. The pyramid level (τ), which dictates the multi-scale decomposition depth, was experimentally determined to be τ = 3 [27]. This choice effectively balances image clarity and color fidelity, as higher levels like τ = 4 or τ = 5 were observed to cause severe color distortion [27]. The LRD scheme decomposes enhanced images into high-frequency and low-frequency sub-band images using LP. Pre-fusion enhancement is handled by the Gradient Domain Image Enhancement (GDIE) module, which involves steps like Maximum Local Difference (MLD), interval division, and gradient remapping [27]. GDIE utilizes adaptive high-frequency information lifting coefficient C(i,j) and a remapping function Y2(x(θ,δ)). The enhancement intensity parameters ω1 and ω2 are also critical, with optimal values identified as ω1 = 1.5 and ω2 = 0.3, based on maximizing Mutual Information (MI) and Universal Quality Index (UQI) [27].

For fusion, distinct decision rules are applied based on the decomposed information. For the low-frequency sub-band images (LSIs), the LEM fusion rule is applied. This rule uses a direct addition operation for local pixel sums instead of squaring and calculates LEM based on a 3 × 3 local window with a filtering template λ = [111; 111; 111] [27]. High-frequency information, classified into OD images and NOD images by the Decision Graph Re-decomposition (DGR) algorithm, uses separate fusion rules. The OD fusion rule for redundant information combines MLD and LEM to create Local Decision Maximums (LDMs) for images, using their comparison to form a binary decision graph. For complementary information in NOD images, a simple addition fusion rule is used to sum the non-overlapping domain images from the anatomical and functional sources, effectively reducing color distortion [27]. Finally, to reconstruct the high-frequency sub-band fusion image and eliminate artifacts, an IRS fusion rule is applied, which involves re-planning pixels around the OD and using global decision graphs. The initial MLD calculation, which informs the GDIE, is also performed within a 3 × 3 window by summing pixels in different directions [27]. For full algorithmic details, refer to [27]. 

To measure the fused image quality, various parameters, including Entropy [26], standard deviation (STD) [26], peak signal-to-noise ratio (PSNR) [26], and the structural similarity index measure (SSIM) [26], were utilized. The mathematical formula and definition of these parameters are completely presented in [26]. 

### 2.3. Mask Creation

In the BraTS dataset, for each subject, the glioma tumor subregion annotation includes the non-enhancing/necrotic core (Label 1), peritumoral edematous/infiltrated tissue (Label 2), and Gadolinium-enhancing/-active tumoral regions (Label 4). In this study, the peritumoral edematous/infiltrated tissue (Label 2) regions of each subject were used as regions of interest (ROI) for extracting the radiomic features.

### 2.4. Radiomics Feature Extraction

To extract a comprehensive set of radiomics features from regions of interest of glioma tumors, PyRadiomics (https://pyradiomics.readthedocs.io/en/latest/, Accessed on 10 January 2024) was used [28]. The PyRadiomics framework allows for the standardized and reproducible extraction of a vast array of quantitative features through the configuration of three distinct components: feature classes, image types, and extraction settings. In this study, feature extraction was configured to derive features from two image types: the ‘Original’ image and ‘Wavelet’ decomposition, which applies a filter to highlight textural information at different frequencies. From these image types, we extracted a comprehensive set of feature classes, including Shape, First-order Statistics, Gray-Level Cooccurrence Matrices (GLCMs), Gray-Level Dependence Matrices (GLDMs), Gray-Level Run Length Matrices (GLRLMs), Gray-Level Size Zone Matrices (GLSZMs), and Neighborhood Gray-Tone Difference Matrix (NGTDM) features of the ROI. Key extraction settings, including a bin width = 25, interpolator = ‘sitkBSpline’, and ‘resampledPixelSpacing’, were used. For a technical reference and computational implementation of all PyRadiomics-extracted radiomic features, see [28].

### 2.5. Feature Selection

To mitigate the impact of feature magnitude differences, we applied MinMax scaling normalization, rescaling all features to a uniform range between 0 and 1. The Boruta [29] was employed as a feature selection method for dimensionality reduction, preserving maximal data variance in the reduced feature space. The Boruta algorithm is an all-relevant feature selection method that identifies the complete set of potentially predictive variables [29]. The method operates by systematically comparing original features against artificially generated shadow features (random permutations of the original data) through an iterative process. Using a statistical testing framework, it classifies features as relevant only when they demonstrate significantly higher importance than the maximum shadow feature importance across multiple iterations, ensuring robust selection against random noise [29]. To train the Boruta algorithm for feature selection purposes, the XGBoost classifier was used.

### 2.6. Classification

In this study, we focused on the glioma grade (LGG/HGG) classification task using radiomics features from peritumoral edema regions in MRI and fused images. The classification task was performed using the Tree-based Pipeline Optimization Tool (TPOT) [30].

TPOT (https://epistasislab.github.io/tpot/latest/, Accessed on 10 January 2024) is an open-source Python (V= 3.10) library for Automated Machine Learning (AutoML). This evolutionary algorithm-based AutoML system evaluated a comprehensive space of classifiers, including tree-based methods (Random Forest (RF), Gradient Boosting (GB), XGBoost), linear models (Logistic Regression (LR), SVM with linear/rbf kernels), distance-based classifiers (KNN), and probabilistic approaches (Naive Bayes (NB)). It uses genetic programming to explore and optimize pipeline structures and hyperparameters [31].

To address the class imbalance issue in the dataset, the “class-weight” method, which adjusts the loss function to penalize misclassification of the minority class more heavily, was applied during the model’s training.

### 2.7. Performance Evaluation

To comprehensively assess the predictive performance of the models for glioma grade classification, we employed multiple established evaluation metrics: accuracy (measuring overall correctness), precision (positive predictive value), recall (sensitivity), and F1-score (harmonic mean of precision and recall). Also, we utilized receiver operating characteristic (ROC) curves with area under the curve (AUC) quantification. Model performance was rigorously evaluated using 10-fold cross-validation to ensure robustness and mitigate overfitting, with all reported metrics representing the average performance across all folds.

## 3. Results

**Figure 2:**
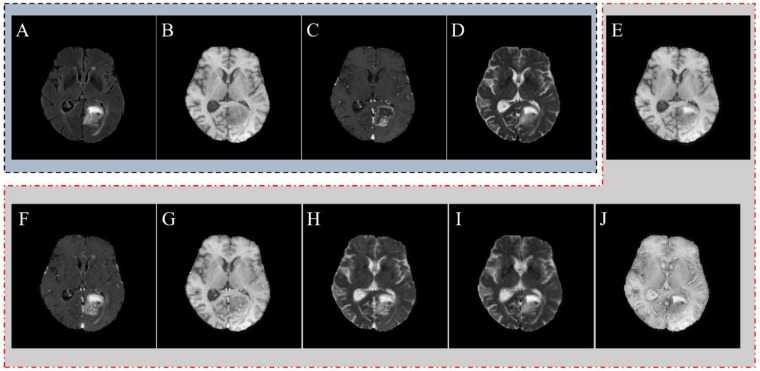
Original MRI and fused images of a high-grade glioma (HGG) subject in the BraTS dataset. (**A**–**D**) original MRI image weights, (**E**–**J**) fused images with the LRD medical image fusion algorithm. (**A**)—FLAIR. (**B**)—T_1_, (**C**)—T_1_Gd. (**D**)—T_2_, (**E**)—T_1_+FLAIR. (**F**)—T_1_Gd+FLAIR. (**G**)—T_1_Gd+T_1_. (**H**)—T_1_Gd+T_2_. (**I**)—T_2_+FLAIR. (**J**)—T_2_+T_1_.

**Figure 3:**
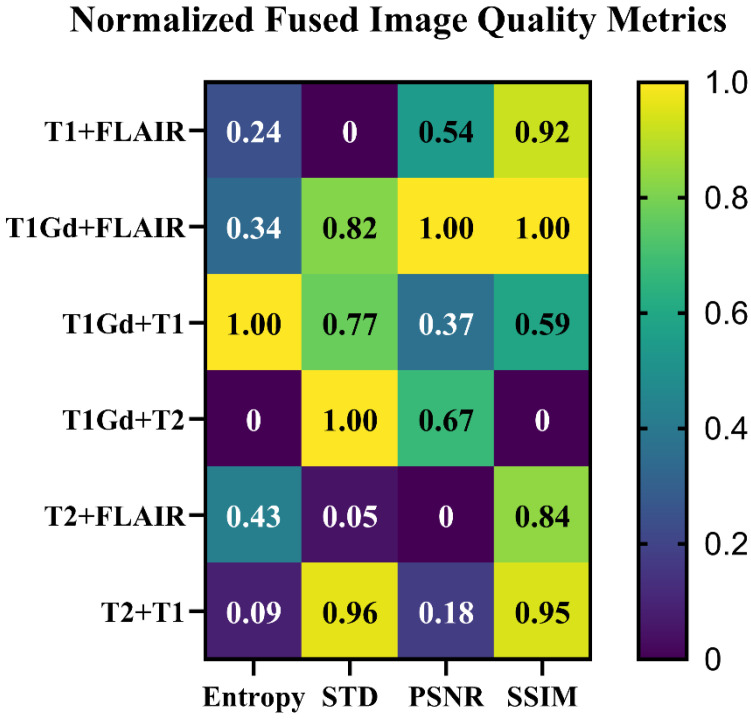
Heatmap of normalized image quality metrics in fused MRI images with the LRD medical image fusion method. Abbreviations: STD: standard deviation, PSNR: peak signal-to-noise ratio, SSIM: structural similarity index measure.

A total of two subjects in the BraTS 2023 dataset were excluded from the study due to image artifact issues (such as Gibbs Ringing (Truncation Artifact)) and edema labeling issues by an MRI specialist. From the BraTS 2023 dataset, 400 glioma cases were included in this study for radiomic feature extraction, with a distribution of 324 (81%) HGGs and 76 (19%) LGGs. To provide fused MRI images for each subject, the LRD medical image fusion method was employed. Figure 2 presents the results obtained from the LRD medical image fusion and original MRI image weights of an HGG subject. The first set of analyses calculated the image quality of fused MRI image weights, and the results are presented as a heatmap in Figure 3 across different fused images (T_1_+FLAIR, T_1_Gd+FLAIR, T_1_Gd+T_1_, T_1_Gd+T_2_, T_2_+FLAIR, T_2_+T_1_). For optimal heatmap visualization of multi-scale metrics with different value ranges (SSIM: [0,1], PSNR: [20–50 dB], Entropy: [0–8 bits], STD: [0–255]), we normalized all values to a standard scale [0,1], allowing clear pattern recognition across all parameters.

**Figure 4:**
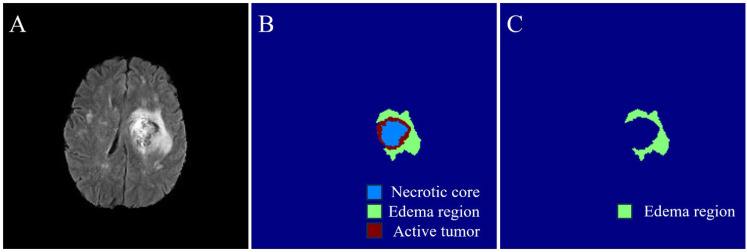
Edema mask creation as a region of interest (ROI) for the radiomic feature extraction process. (**A**)—Sample FLAIR MRI image weight of an HGG subject. (**B**)—Corresponding mask with three different annotated regions: necrotic, edema, and active tumor cell regions. (**C**)—Corresponding created edema region annotated mask used for feature extraction.

Figure 4 illustrates the process of annotating an edema region in an HGG subject’s slice and generating masks for radiomic feature extraction from its corresponding mask. The extracted edema region masks were used as ROIs for the radiomic feature extraction process.

The extracted features from the edema region of glioma tumors in this study included 14 Shape features derived from masks and 93 image-based features, which are composed of 18 First-order, 24 GLCM, 14 GLDM, 16 GLRLM, 16 GLSZM, and 5 NGTDM features. Also, 744 features were extracted from wavelet sub-band images. Consequently, the feature set for each MRI image, as well as the fused images, included 851 radiomic features. The results obtained from the Boruta feature selection method across MRI image weights (FLAIR, T_1_, T_1_Gd, T_2_) and fused images (T_1_+FLAIR, T_1_Gd+FLAIR, T_1_Gd+T_1_, T_1_Gd+T_2_, T_2_+FLAIR, T_2_+T_1_) with the LRD medical image fusion method are presented in Table 1.

**Figure 5:**
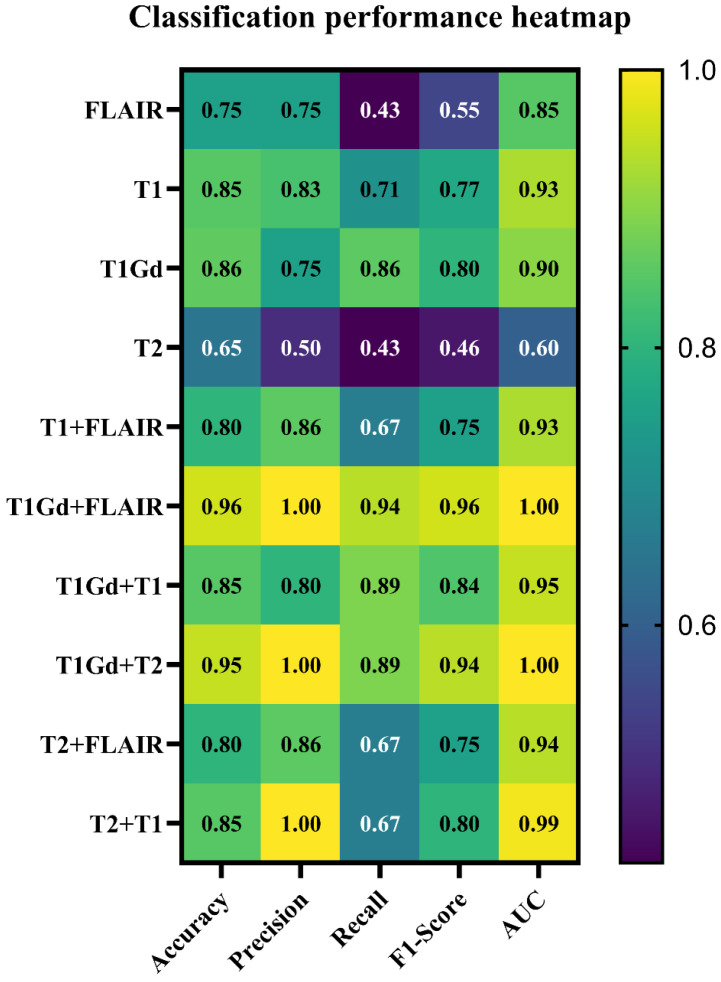
The top-performing trained models’ performance heatmap for glioma grade classification (LGG/HGG classification) with different image inputs. Abbreviation: AUC: area under the curve.

**Figure 6:**
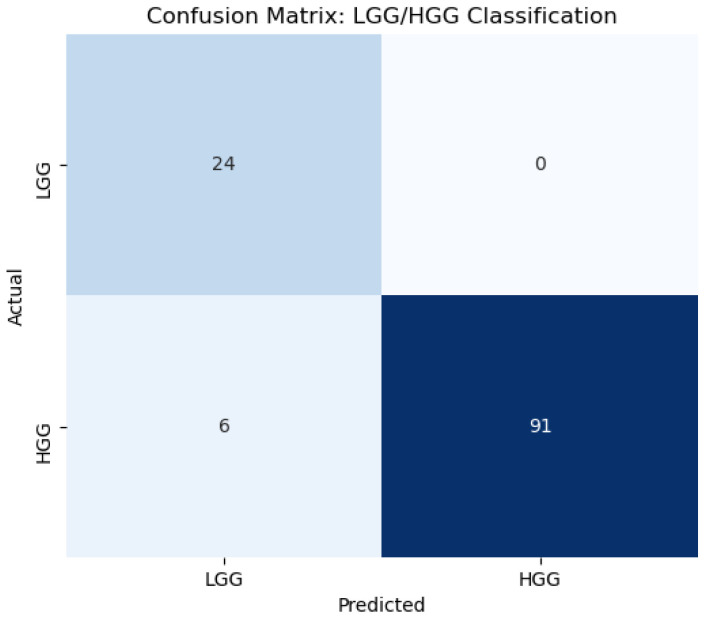
The confusion matrix of a tuned Stochastic Gradient Descent (SGD) classifier trained with a T_1_Gd+FLAIR fused image for glioma grade LGG/HGG classification.

The TPOT automated pipeline results are summarized in Table 2. The optimized and top-performing classifier for LGG/HGG classification across different MRI image weights and fused MRI images is listed in this table. The performance heatmap of top-performing trained classifiers for glioma grade classification (LGG/HGG classification) with different MRI image weights and MRI fused images is shown in Figure 5. In Figure 5, there is a clear improvement in glioma grade classification when we use fused MRI images as input images compared to MRI image weights. What is interesting in the data in Table 2 and Figure 5 is that with a tuned Stochastic Gradient Descent (SGD) classifier and trained with T_1_Gd+FLAIR (fused T_1_Gd with FLAIR images) with parameters in Table 2, it achieves the highest performance (Accuracy = 0.96, Precision = 1, Recall = 0.94, F1-Score = 0.96, and AUC = 1) for LGG/HGG glioma grade classification. The confusion matrix of the top-performing classifier and image combination (tuned SGD and training with T_1_Gd+FLAIR fused image) for glioma grade classification is shown in Figure 6.

## 4. Discussion

As mentioned in the introduction, glioma grade classification is a crucial component in neurology, playing a key role in patient treatment management and decision-making. In this study, we investigate how image fusion and radiomic features of peritumoral edema regions in MRI fused images affect the glioma grade classification. By analyzing peritumoral edema radiomic features rather than tumor core and active tumor regions, we identified a spatially distinct imaging pattern in fused images that may serve as a biomarker for non-invasive glioma grade classification. We fused MRI image weights with the LRD medical image fusion method and extracted radiomic features from peritumoral edema regions in MRI image weights (FLAIR, T_1_, T_1_Gd, T_2_) and fused MRI images (T_1_+FLAIR, T_1_Gd+FLAIR, T_1_Gd+T_1_, T_1_Gd+T_2_, T_2_+FLAIR, T_2_+T_1_), focusing on glioma grade LGG/HGG classification. These features were selected by Boruta, and TPOT then classified the selected features for the LGG/HGG glioma grade classification task. This study critically examined how the peritumoral edema region image features in fused MRI images, as influenced by the LRD medical image fusion, affect glioma tumor grade classification performance.

In the current study, comparing SSIM, PSNR, Entropy, and STD values as image quality metrics revealed that the LRD medical image fusion is a powerful method for producing high-quality images. These values and results are consistent with those of previous studies [23,24,25]. Additionally, these findings further support the idea that LRD medical image fusion can create high-quality fused images.

The result of this study shows that ML models using radiomic features of peritumoral edema can be used for glioma grade classification, and these results match those observed in earlier studies [15]. These findings further support the potential use of peritumoral edema region radiomic features as a non-invasive, fast, and accurate method for LGG/HGG glioma grade classification. These results can be explained through tumor-microenvironment interactions and behavior in LGGs and HGGs. The peritumoral edema regions exhibit fundamentally distinct characteristics between glioma grades. While LGGs demonstrate infiltrative edema resulting from tumor cell dispersion, HGGs generate vasogenic edema through VEGF-mediated disruption of the blood–brain barrier (BBB) [32,33].

In this section, we evaluate how MRI radiomic features and image fusion techniques enhance glioma grade classification performance. It is interesting to note that trained models with fused MRI image radiomic features, on average, outperformed those using MRI image radiomic features. This result may be explained by the fact that different MRI weightings provide complementary information and data, and image fusion techniques integrate these multimodal data into a unified representation, yielding enriched radiomic features that significantly improve the accuracy of glioma grade classification. This finding aligns with prior studies [24,25,26] that demonstrate the fusion of multimodal MRI sequences generates fused images that preserve complementary data from both sources, where the resulting signal intensity in the fused images enables accurate glioma grade classification. The findings of trained models using MRI image weights (FLAIR, T_1_, T_1_Gd, T_2_) radiomic features are consistent with those of Azemi G and Di Ieva A [14], who utilized MRI image weight radiomic features of edema regions for glioma grade classification.

The most interesting finding was that fine-tuned SGD models trained with radiomic features of T_1_Gd+FLAIR fused images consistently outperformed those using all other fused images in the current study. This result may be explained by the fact that while T_1_Gd imaging captures tumor proliferation and vascular enhancement patterns [34], FLAIR sequences highlight pathological tissue alterations through prolonged T_2_ relaxation times, particularly in regions of edema or inflammation [35]. The fusion of T_1_Gd and FLAIR MRI image weights combines pathological information, integrating proliferation data with edema or inflammation data, to yield a fused image with enhanced diagnostic utility for glioma characterization in our study. An important finding in the clinical use of our method was that a limited number of misclassifications were observed, primarily concerning the misclassification of HGG as LGG in the confusion matrix (Figure 6). Despite the infrequency of these misclassifications, their potential clinical impact is severe, warranting a cautious interpretation of the results and highlighting an important area for future model improvement for clinical use.

The most important limitation of this study lies in the fact that LGG cases are a relatively small part of the dataset population, and the dataset is homogeneous. Expanding the dataset to include different tumor subtypes and the LGG population would help generalize and validate the study results. Additionally, using external validation could provide greater generalizability and reliability for the model’s predictions. An issue that was not addressed in this study was the segmentation of peritumoral edema. In the clinic, segmentation of these regions for feature extraction is a time-consuming stage. It is recommended that further research be undertaken for automatic peritumor edema segmentation, such as using a trained U-Net. Previous studies [36,37] have shown that diffusion-weighted imaging (DWI) and diffusion tensor imaging (DTI) have a high ability to detect cognitive diseases and Alzheimer’s disease, which indicates the high information content of these images. It is suggested that in the future, the radiomic features of these images can be used.

## 5. Conclusions

This is the first time that radiomic features of peritumoral edema regions in fused MRI images have been used for glioma grade classification. Our analysis revealed that radiomic features extracted from peritumoral edema in fused MRI images using the LRD medical image fusion method exhibit distinct patterns that correlate with glioma tumor grade. In general, therefore, it seems that the radiomic features from peritumoral edema regions and their interaction with surrounding tissue provide valuable data for glioma grading. These findings demonstrate that peritumoral radiomic features in fused images can serve as a non-invasive biomarker for assessing tumor behavior and microenvironment interaction, which are important for glioma grade. Our method demonstrates potential clinical utility for non-invasive glioma grading; however, the translation of this data into routine practice requires further validation in multi-center trials and the development of user-friendly, certified software to ensure seamless integration into the clinical workflow.

The results of this investigation demonstrate that Boruta is a powerful feature extraction method and TPOT is a robust AutoML classifier in glioma grade classification using radiomic features of peritumoral edema regions.

## Figures and Tables

**Table 1:** The result of the Boruta method is a feature selection method. The top-ranked feature and selected features for each MRI image weight and fused image are presented.

	Image	Selected Features
Original MRI images	FLAIR	Maximum 3D Diameter, JointEntropy, Correlation.1, DifferenceAverage.1, Imc1.1, Imc2.1, MCC.1, Complexity.2, Maximum.3, 10Percentile.4, DifferenceEntropy.5, LowGrayLevelEmphasis.7
	T_1_	SmallAreHighGrayLevelEmphasis, InterquartileRange.1, Median.2, Imc1.2, Kurtosis.3, 10Percentile.4, Kurtosis.5, SmallAreaLowGrayLevelEmphasis.5, MeanAbsoluteDeviation.6
	T_1_Gd	SmallDependenceLowGrayLevelEmphasis, Mean.1, Median.1, Mean.2, Idmn.2
	T_2_	10Percentile.1, RootMeanSquared 1, MCC.1, Uniformity.2, Maximum.3, Contrast.6, DifferenceAverage.3, Imc1.6, Kurtosis.7
Fused images	T_1_+FLAIR	Idmn, Contrast.2, Imc2.1, GrayLevelVariance.6, Skewness.3, Idn.3, Imc1.7
	T_1_Gd+FLAIR	DependenceVariance, 90Percentile.1, Median.1, Correlation.1, DifferenceVariance 1, Imc2.1, MCC.1, MaximumProbability.1, GrayLevelNonUniformityNormalized.3, Mean.2, Imc1.2, MaximumProbability.3, Imc1.4, MaximumProbability.5, MCC.7
	T_1_Gd+T_1_	Clustershade, Contrast, DependenceVariance, 10Percentile.1, Maximum.1, Skewness.1, Cnotrast.2, Idm.1, Imc.1, Mean.2, MaximumProbability.2, Imc2.3
	T_1_Gd+T_2_	Flatness, Kurtosis, LowGrayLevelZoneEmphasis, Entropy.1, Mean.1, Clustershade.1, DifferenceVariance.1, DependenceEntropy.1, DependenceNonUniformityNormalized.1, GrayLevelVariance.3, GrayLevelVariance.4, Entropy.2, Mean.2, Imc1.2, SmallAreaEmphasis, Correlation.3, Imc2.3, MaximumProbability.3, Kurtosis, Mean4, Autocorrelation5, Imc1.6, HighGrayLevelZoneEmphasis.6
	T_2_+FLAIR	DifferenceVariance.1, ZoneEntropy.1, Idmn.3, Skewness.4, DependenceEntropy.5
	T_2_+T_1_	SurfaceVolumeRatio, Imc.1, HighGrayLevelZoneEmphasis, 10Percentile.1, 90Precentile.1, Entropy.1, MeanAbsoluteDeviation.1, Clustershade.1, Contrast.2, Imc2.1, GrayLevelNonUniformityNormalized.2, DifferenceAverage.2, HighGrayLevelZoneEmphasis.2, SmallAreaEmphasis.2, SmallAreaLowGrayLevelEmphasis.2, ZoneEntropy.2, ZonePercentage.2, Complexity.2, MaximumProbability.3, MeanAbsouluteDeviation.4, DependenceVariance.4, GrayLevelNonUniformityNormalized.9, ZoneEntropy.4, MaximumProbability.7

**Table 2:** The results of the Tree-based Pipeline Optimization Tool (TPOT) method for choosing the best machine learning model for the HGG/LGG classification problem with different MRI image weights and fused MRI images by the LRD medical image fusion method. Abbreviation: SGD: Stochastic Gradient Descent, MLP: Multi-Layer Perceptron, KNN: k-Nearest Neighbors.

Image	Best Classifier	Parameters
FLAIR	Gradient Boosting	Learning_rate = 0.01, max_depth = 8, max_features = 1.0, min_samples_leaf = 11, min_samples_split = 14, n_estimators = 100, subsample = 0.6501
T_1_	Gradient Boosting	Learning_rate = 0.5, max_depth = 2, max_features = 0.1, min_samples_leaf = 4, min_samples_split = 20, n_estimators = 100, subsample = 0.55
T_1_Gd	Bernoulli Naïve Bayes	Alpha = 100, fit_prior = False
T_2_	SGD Classifier	Alpha = 0.01, eta = 0.1, fit_intercept = False, l1_ratio = 0.25, learning_rate = `constant`, penalty = `elasticnet`, power_t = 10
T_1_+FLAIR	MLP	Alpha = 0.0001, learning_rate_init = 0.1
T_1_Gd+FLAIR	SGD Classifier	Alpha = 0.001, eta = 0.01, fit_intercept = True, l1_rati = 0.25, learning_rate = `invscaling`, penalty = `elasticnet`, power_t = 0.1
T_1_Gd+T_1_	Gradient Boosting	Learning_rate = 0.1, max_depth = 3, max_features = 0.95, min_samples_leaf = 9, min_samples_split = 13, n_estimators = 100, subsample = 0.7
T_1_Gd+T_2_	Gradient Boosting (Stacking Estimator)	Learning_rate = 0.1, max_depth = 10, max_features = 0.5, min_samples_leaf = 14, min_samples_split = 10, n_estimators = 100, subsample = 0.5
	MLP (Stacking Estimator)	Alpha = 0.001, learning_rate_init = 1
	KNN (Classifier)	N_neighbors = 33, p = 1, weights = `distance`
T_2_+FLAIR	KNN	N_neighbors = 39, p = 1, weights = `uniform`
T_2_+T_1_	MLP	Alpha = 0.0001, learning_rate_init = 0.1

## Data Availability

The datasets used in this study are available at (brats@cbica.upenn.edu, http://braintumorsegmentation.org, 10 January 2024). The analyzed data and models are available from the corresponding author upon reasonable request.
